# A branching process model for the analysis of abortive colony size distributions in carbon ion-irradiated normal human fibroblasts

**DOI:** 10.1093/jrr/rrt129

**Published:** 2014-02-04

**Authors:** Tetsuya Sakashita, Nobuyuki Hamada, Isao Kawaguchi, Takamitsu Hara, Yasuhiko Kobayashi, Kimiaki Saito

**Affiliations:** 1 Microbeam Radiation Biology Group, Japan Atomic Energy Agency (JAEA), 1233 Watanuki, Takasaki, Gunma 370-1292, Japan; 2 Radiation Safety Research Center, Nuclear Technology Research Laboratory, Central Research Institute of Electric Power Industry (CRIEPI), 2-11-1 Iwado-kita, Komae, Tokyo 201-8511, Japan; 3 Regulatory Science Research Program, Research Center for Radiation Protection, National Institute of Radiological Sciences (NIRS), 4-9-1, Anagawa, Inage, Chiba, 263-8555, Japan; 4 Division of Translational Research for Drug Discovery, Fukushima Medical University, 1 Hikarigaoka, Fukushima, Fukushima 960-1295, Japan; 5 Fukushima Environmental Safety Center, JAEA, 2-2-2 Uchisaiwai, Chiyoda, Tokyo 100-0011, Japan

**Keywords:** branch dynamics, delayed reproductive cell death, non-targeted effect, ionizing radiation

## Abstract

A single cell can form a colony, and ionizing irradiation has long been known to reduce such a cellular clonogenic potential. Analysis of abortive colonies unable to continue to grow should provide important information on the reproductive cell death (RCD) following irradiation. Our previous analysis with a branching process model showed that the RCD in normal human fibroblasts can persist over 16 generations following irradiation with low linear energy transfer (LET) γ-rays. Here we further set out to evaluate the RCD persistency in abortive colonies arising from normal human fibroblasts exposed to high-LET carbon ions (18.3 MeV/u, 108 keV/µm). We found that the abortive colony size distribution determined by biological experiments follows a linear relationship on the log–log plot, and that the Monte Carlo simulation using the RCD probability estimated from such a linear relationship well simulates the experimentally determined surviving fraction and the relative biological effectiveness (RBE). We identified the short-term phase and long-term phase for the persistent RCD following carbon-ion irradiation, which were similar to those previously identified following γ-irradiation. Taken together, our results suggest that subsequent secondary or tertiary colony formation would be invaluable for understanding the long-lasting RCD. All together, our framework for analysis with a branching process model and a colony formation assay is applicable to determination of cellular responses to low- and high-LET radiation, and suggests that the long-lasting RCD is a pivotal determinant of the surviving fraction and the RBE.

## INTRODUCTION

The relative biological effectiveness (RBE) varies with the linear energy transfer (LET) of ionizing radiation (IR). Compared with low-LET photons like X-rays or γ-rays, high-LET heavy ions cause more dense ionization along trajectories and more complex clustered DNA damage, thereby being more effective at inactivating a cellular clonogenic potential [1–5].

**Figure 1:**
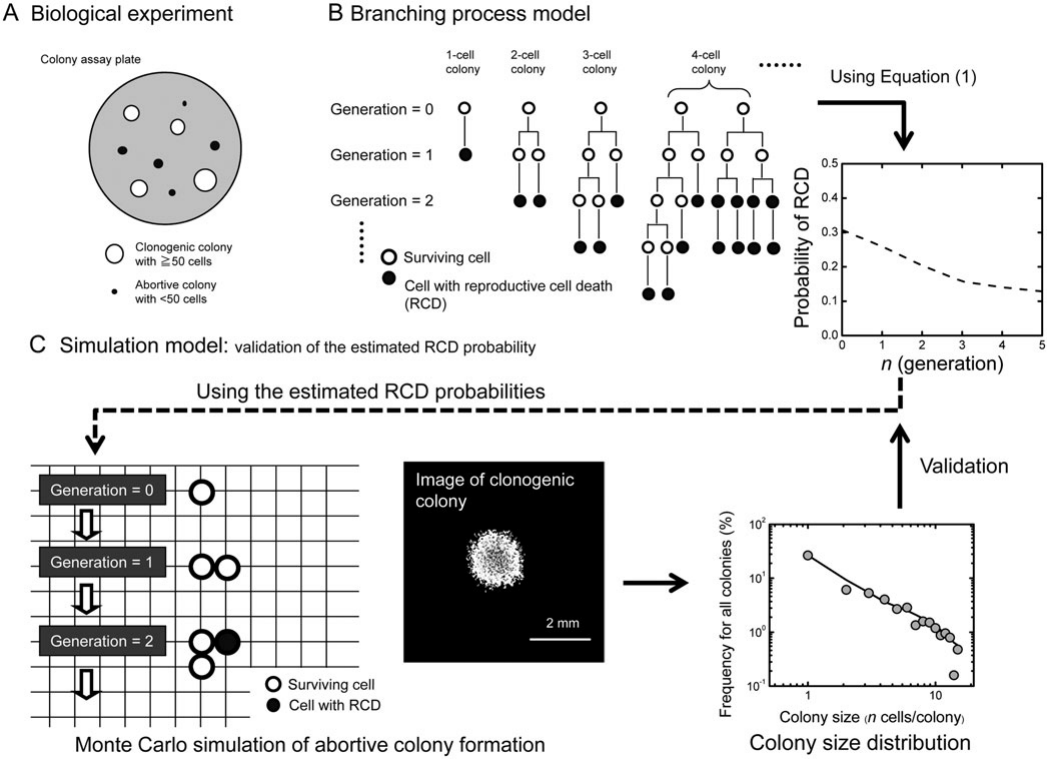
Flow chart of the present study. (**A**) Biological experiments. The colony formation assay was conducted on the plate, then the number of resulting surviving colonies with 50 cells or more (open circles) and abortive colonies with less than 50 cells (closed circles) as well as the number of cells in each colony were counted. (**B**) The analysis was performed with a branching process model. Examples of cell lineage and the probability profile of reproductive cell death (RCD) estimated using Equation (1) are presented. (**C**) Monte Carlo simulation of abortive colony formation to evaluate the validity of the estimated RCD probability. Representative images of the *in silico* clonogenic colony and the simulated abortive colony size distribution are shown.

Clonogenicity is the ability of a cell to form a colony, and alters in response to a variety of internal and external stimuli. In a colony formation assay, clonogenic colonies each comprising 50 cells or more are generally evaluated as survivors, while abortive colonies that contain less than 50 cells are regarded as non-survivors because of their failure to continue to grow (Figure. 1A). In contrast to the case for clonogenic colonies, the fraction of abortive colonies increases with increasing dose in normal human fibroblasts [6], suggestive of a systematic rule in the production kinetics of abortive colonies. Our previous work showed that the abortive colony size distribution after low-LET exposure can follow a linear relationship on the log–log plot, along with a systematic rule based on the branching processes [7]. A question arises herein as to how more cytocydal high-LET radiation affects the production kinetics of the abortive colonies. The present study was therefore undertaken to investigate the abortive colonies arising from normal human fibroblasts exposed to high-LET radiation.

The probability of reproductive cell death (RCD) is an important parameter for understanding the clonogenic kinetics, and our previous work showed persistence of the RCD probability multiple generations after γ-irradiation [7]. Here we show the RCD probability in the progeny of high- LET-irradiated human fibroblasts, as well as the RBE estimated based on the branching process model. Moreover, we analyze the secondary colony formation [3], which is important for understanding the RCD probability in the late phase of the clonogenic kinetics. Our present study provides insights into the production kinetics of abortive and clonogenic colonies formed following exposure to low- and high-LET radiation.

## MATERIALS AND METHODS

### Cell culture, irradiation and colony formation assay

For all experiments, AG01522D primary normal human diploid foreskin fibroblasts purchased from the Coriell Cell Repositories at the Coriell Institute for Medical Research (Camden, NJ, USA) were used. Cells were exposed to carbon ions (18.3 MeV/u, 108 keV/µm) delivered from the azimuthally-varying-field cyclotron installed at the Takasaki Ion accelerators for Advanced Radiation Application (TIARA) facility of the Japan Atomic Energy Agency (JAEA). The absorbed dose was calculated according to the formula: dose (Gy) = 1.6 × 10^−9^ × LET (keV/µm) × fluence (particles/cm^2^) [3]. Cell cultures and colony formation were carried out as previously described [3], and colonies containing ≥ 2 cells were counted. Of these, colonies with 2–49 cells are referred hereafter to as abortive colonies, whereas those with 50 cells or more are referred to as clonogenic colonies. The summarized data on the analysis of colonies and surviving fractions have been previously reported [6], and the original detailed datasets were used in this study.

### Branching process model

Irradiated cells should undergo RCD or proliferation, and form an abortive colony through a complicated cell lineage expressed as a branch tree (Figure 1B). To analyze the production kinetics of abortive colonies, we introduced *P*_*1*_ (the probability of the RCD) and *P*_*2*_ (the probability of proliferation) at each branch point, and the *f* (occurrence frequency of abortive colonies with *n* cells) was calculated as follows:

(1) $$f_n = P_2 \times \sum\limits_{\,j = 0}^{n - 2} {\left( {\,f_{1 + j} \times f_{n - j - 1} } \right),\left( {n \ge 2} \right)} $$

Where *f_1_* = *P_1_* and *P_2_* = 1 − *P_1_*.

The frequency of 1-cell colonies was estimated from that of 2-cell colonies, assuming a similar probability of RCD for 1- and 2-cell colonies as previously described [7]. The phrase ‘frequency for all colonies’ is used to refer to the distribution of all colonies (abortive colonies including a 1-cell colony plus clonogenic colonies), and the word ‘frequency’ was used for the distribution of colonies with ≥ 2 or more cells. Moreover, we evaluated *P*_*1*_ (*g*) (= 1 − *P*_*2*_ (*g*)) at generation *g* < 6 from the regression curve for the abortive colony size distribution with a 95% confidence limit, as previously described [7]. We used the linear interpolation for *P*_*1*_ (*g* ≥ 6), where *P*_*1*_ (*g* = 16) was set as (1 – *c*) multiplied by *P*_*1*_ (*g* = 5) and *c* was the constant.

### Simulation model

To simulate the colony formation kinetics, we used the Monte Carlo method based on the branching processes with the estimated RCD probabilities, i.e. *P*_*1*_ (*g*) [7] (Figure 1C). The simulation model considered 2D colony expansion, because a colony derived from AG01522D cells is a flat, single monolayer [8]. A cell was located on the 2D calculation grid point, of which size was set at 31.6 µm × 31.6 µm, i.e. one cell area of ∼ 1000 µm^2^. When a cell has no free space in the eight adjacent grid points it cannot divide into two cells. We used the experimentally determined doubling time of 20 h [3] and calculated the fate of irradiated cells at each doubling according to *P*_*1*_ (*g*). A clonogenic colony was formed about 13 days (16 divisions) after IR [6]. One simulation consisted of 10 000 repetitions of the calculation based on the 1-cell inoculation into the 2D calculation grid, and each calculation condition was evaluated by five simulation sets where the total inoculated cells numbered 50 000.

### Platform

For a simulation, the platform of the NetLogo software [9] was used, which is freely downloadable at http://ccl.northwestern.edu/netlogo/. NetLogo is a java platform that has been used to analyze complex systems, and adopts the multi-agent modeling Logo languages.

### Statistical analysis

Using the ORIGIN computer program (MicroCal Software, MA, USA), the non-linear regression analysis and calculations of 95% confidence limits for the colony size distribution were carried out. A better fit model was selected with the Akaike Information Criterion (AIC) with log-transformed data, except for the estimated frequency of 1-cell colonies determined according to the previously described method [7]. A chi-squared test was used to make statistical comparisons between experimental data and simulated data, and *P* values were corrected using the Bonferroni method.

## RESULTS

### Experimental determination of the size distribution of abortive clones arising from carbon ion-irradiated cells

**Figure 2:**
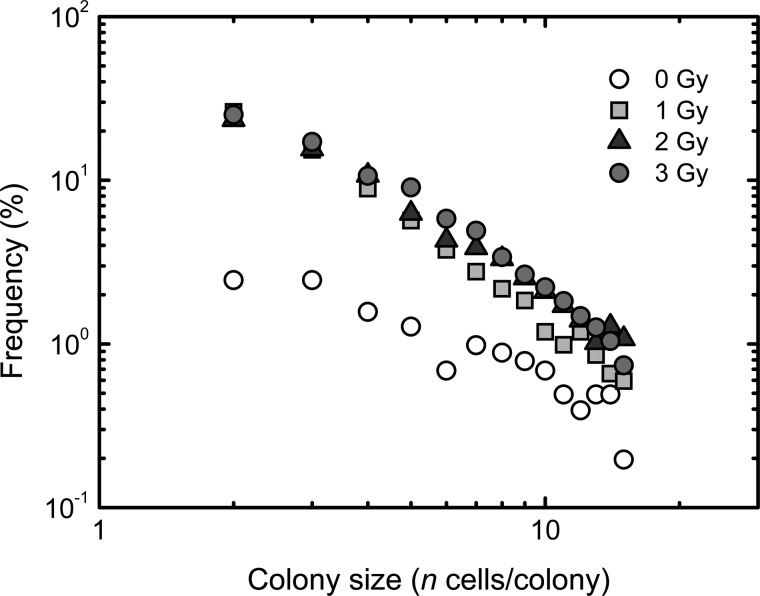
Empirical colony size distribution. Open circles, squares, triangles and gray circles indicate the experimentally determined data at 0, 1, 2 and 3 Gy of carbon ions (18.3 MeV/u, 108 keV/µm), respectively. ‘Frequency’ represents the frequency of a colony with *n* cells in all abortive and clonogenic colonies. The data presented are means of three independent experiments with quadruplicate measurements.

Abortive colonies with < 16 cells derived from normal human fibroblasts showed a linear relationship on the log–log plot following low-LET radiation exposure [7]. On the other hand, high-LET exposure induces more severe damage in a reproductive system than low-LET exposure, and may cause a drastic change in the production kinetics of abortive colonies. Thus, it would be expected that the observations obtained with high-LET radiation would differ from those obtained with low-LET radiation. To examine this in carbon-ion irradiated abortive colonies, we first plotted the colony size distribution on the log–log plot (Fig. 2). The size distribution of abortive colonies arising from carbon ion-irradiated cells followed a linear relationship on the log–log plot (Fig. 2), suggesting that our previously proposed framework for abortive colonies formed following low-LET radiation exposure [7] would be applicable to those following high-LET radiation exposure.

### RCD and its radiogenic excess probabilities

**Figure 3:**
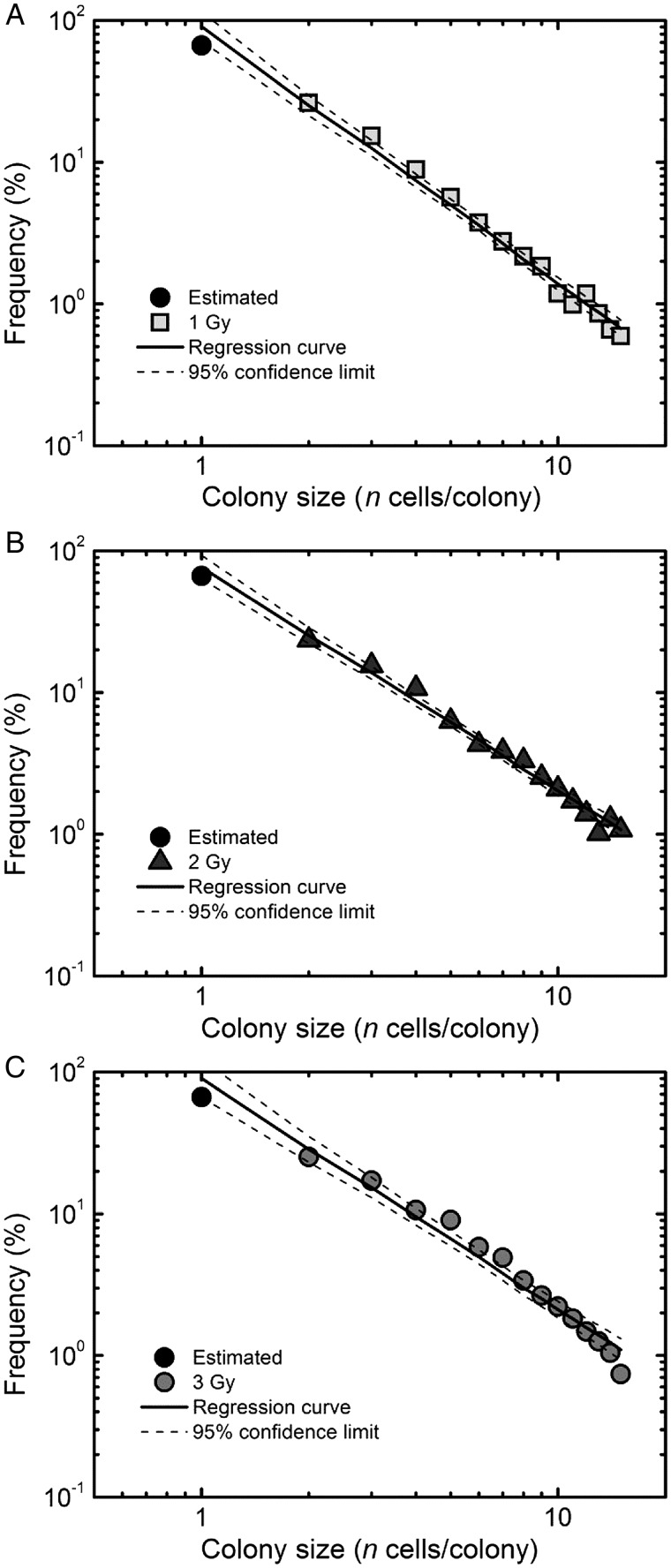
Regression analysis on the log–log plot in the experimentally determined size distribution of abortive colonies at 1, 2 and 3 Gy of carbon ions. The regression analysis was carried out using the experimentally determined frequency of 2-cell colonies and the postulated frequency of 1-cell colonies (solid circles) assuming a similar RCD probability for 1- and 2-cell colonies, as previously described [7]. Solid lines indicate the regression curves, and the dotted lines indicate the curves for 95% confidence limits.

**Figure 4:**
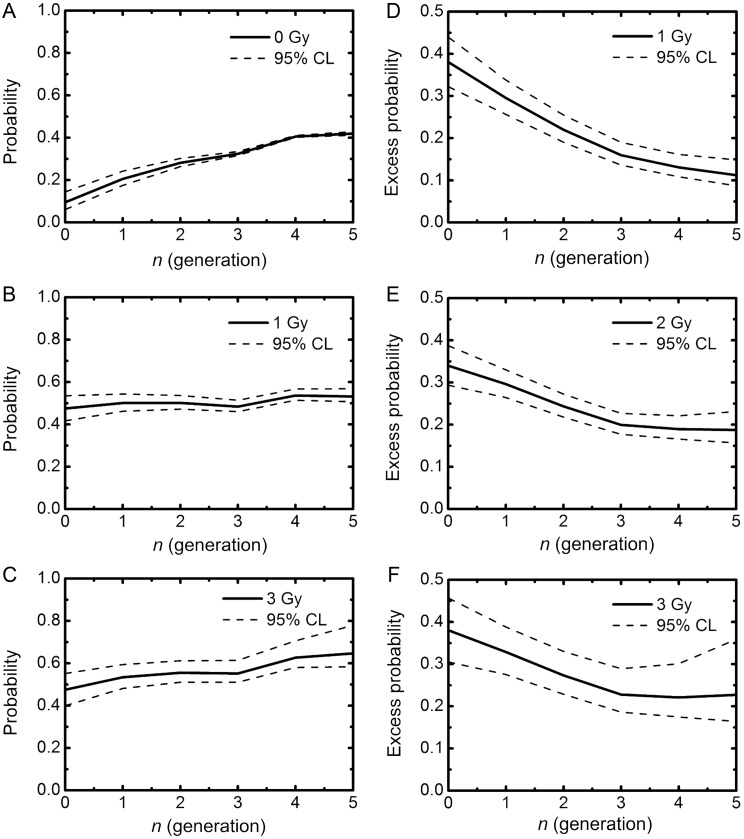
Reproductive cell death probability *P*_*1*_ (0, 1 and 3 Gy of carbon ions) (**A–C**) and the excess *P*_*1*_ (1, 2 and 3 Gy) (**D–F**) at generations 0 to 5. *P*_*1*_ was calculated with Equation 1, using the regression curves in Fig. 2A–C. IR-induced excess *P*_*1*_ was estimated from *P*_*1*_ of abortive colonies derived from non-irradiated cells minus that from irradiated ones. Dotted lines indicate 95% confidence limits.

Our proposed framework allows estimation of the RCD and IR-induced excess RCD probabilities from the abortive colony size distribution following low-LET radiation [7]. With this approach, the frequency of 1-cell colonies was estimated assuming the similar RCD probabilities for 1- and 2-cell colonies. The linear regression analysis with 95% confidence limits on the log–log plot demonstrated the better fit to the datasets (Figure 3A–C; the squared correlation coefficients *R*^*2*^ were 0.988 for 1 Gy, 0.990 for 2 Gy and 0.976 for 3 Gy). Using the log–log regression curve for the experimental datasets (Figure 3A–C), the RCD (*P*_*1*_ (*g*)) and IR-induced excess RCD probabilities at generations 0 to 5 were evaluated from Equation 1. The RCD probability for fibroblasts exposed to 1 Gy of carbon ions demonstrated almost constant values at generations 0 to 5 (Fig. 4B), and was slightly lower than that for 3 Gy at the fifth generation (Fig. 4C). The difference was reflective of the higher excess RCD probability for 3 Gy at the fifth generation (Fig. 4D and F), indicating that the higher the dose the more persistent the RCD in normal human fibroblasts.

### The simulated size distribution of abortive colonies and surviving fraction

**Figure 5:**
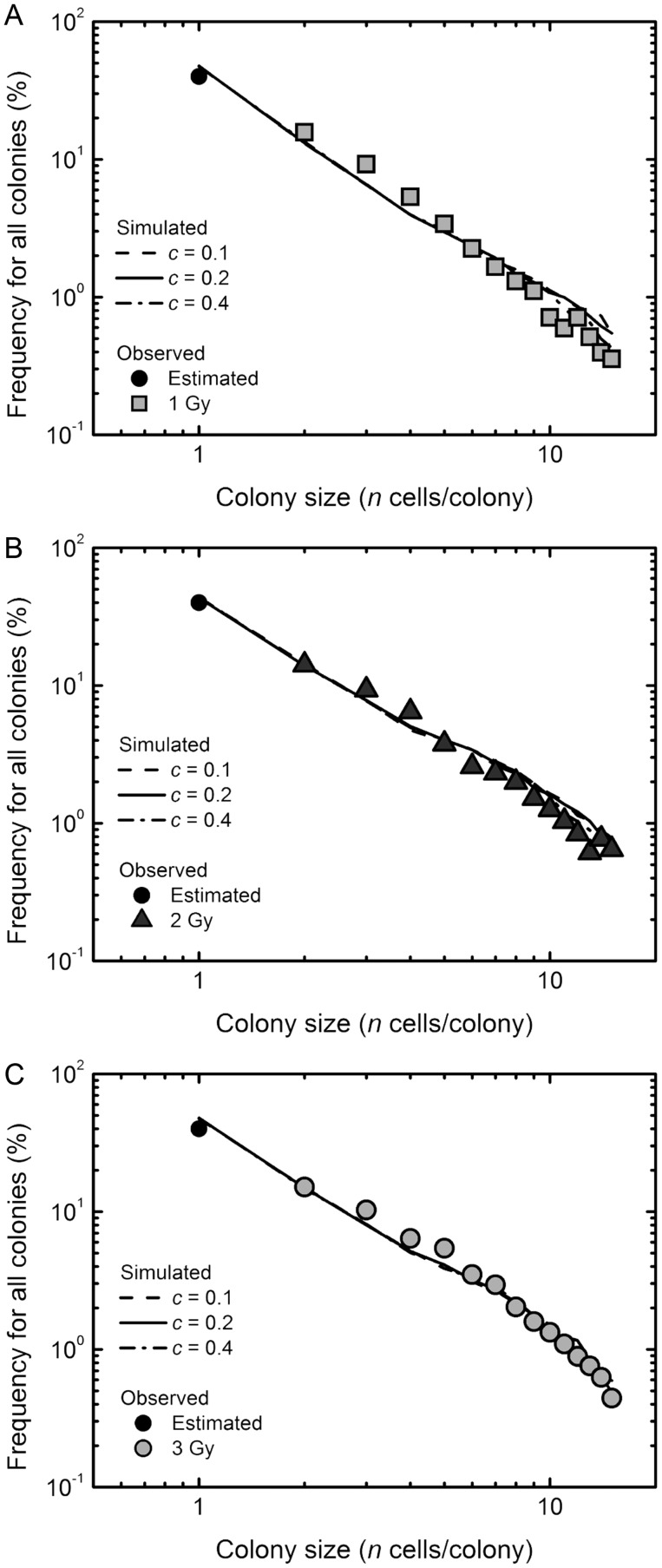
Simulated colony size distribution of abortive colonies arising from carbon ion-irradiated cells. The abortive colony size distributions at 1, 2 and 3 Gy are shown in panels (**A**), (**B**) and (**C**), respectively. Circles demonstrate the experimentally determined colony size, and lines represent the simulated colony size distribution with the specific slope *c* of 0.1, 0.2 or 0.4 using the parameters obtained from the log–log fit.

**Figure 6:**
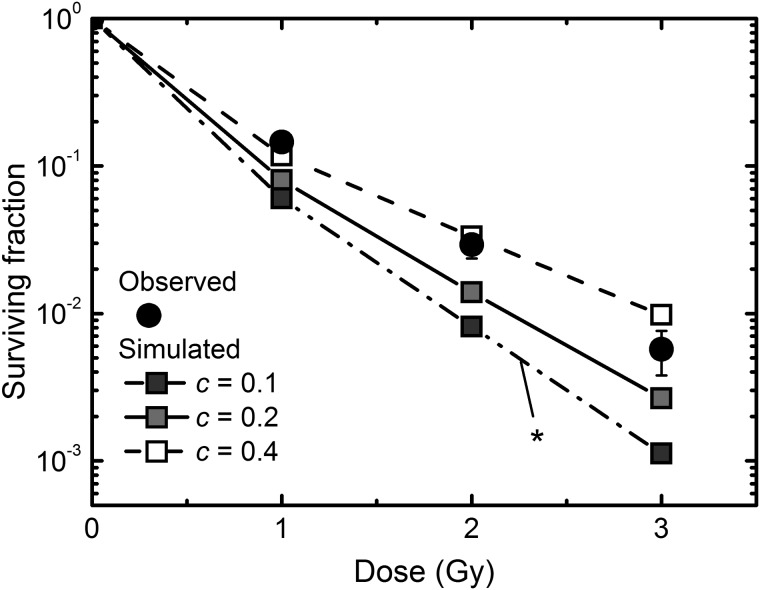
Surviving fraction estimated from the simulated abortive colony formation. Solid circles demonstrate the experimentally determined surviving fraction, and lines with squares represent the simulated surviving fraction with the specific slope *c* = 0.1, 0.2 or 0.4 using the parameters obtained from the log–log fit. Error bars represent standard errors of the mean for the solid circles. An asterisk indicates a significant difference (*P* < 0.05) between observed and simulated dose–response curves tested by the chi-squared test with the Bonferroni correction.

To verify the estimated RCD probability, we simulated the size distribution of abortive colonies using the 2D colony expansion model based on the branching processes, and the surviving fraction was also evaluated for the long-lasting RCD. The simulated size distribution of abortive colonies formed after exposure to 1, 2 and 3 Gy of carbon ions was in good agreement with the observed ones (Fig. 5A–C). Here we assumed the simple linear model for the RCD probability: namely, *P*_*1*_ (*g* = 16) was set as (1 – *c*) multiplied by *P*_*1*_ (*g* = 5), and the values between *P*_*1*_ (*g* = 5) and *P*_*1*_ (*g* = 16) were interpolated as previously reported [7]. There was little detectable difference at *c* values of 0.1, 0.2 and 0.4 (Fig. 5A–C). As is evident from Table 1, the lowest AIC value was obtained at *c* = 0.4 for the three dose points tested, demonstrating the validity of the estimated RCD probability. In contrast, the simulated surviving fraction depended on *c* values (Fig. 6), and the best fit was obtained at *c* = 0.4 (Fig. 6). These findings support our previous findings for low-LET radiation [7] that the short-term RCD is critical to the abortive colony size distribution, and that the long-lasting RCD is important for the surviving fraction.

**Table 1:** Changes in the AIC values depending on the *c* values in the abortive colonies arising from carbon ion-irradiated cells, where *c* is the parameter for the simple linear model

AIC			
Type	Dose (Gy)		
	1	2	3
*c* = 0.1	7.975	− 0.702	− 8.217
*c* = 0.2	5.503	− 2.724	− 13.287
*c* = 0.4	− 2.113	− 7.990	− 20.891

### RBE and excess RCD probability

**Figure 7:**
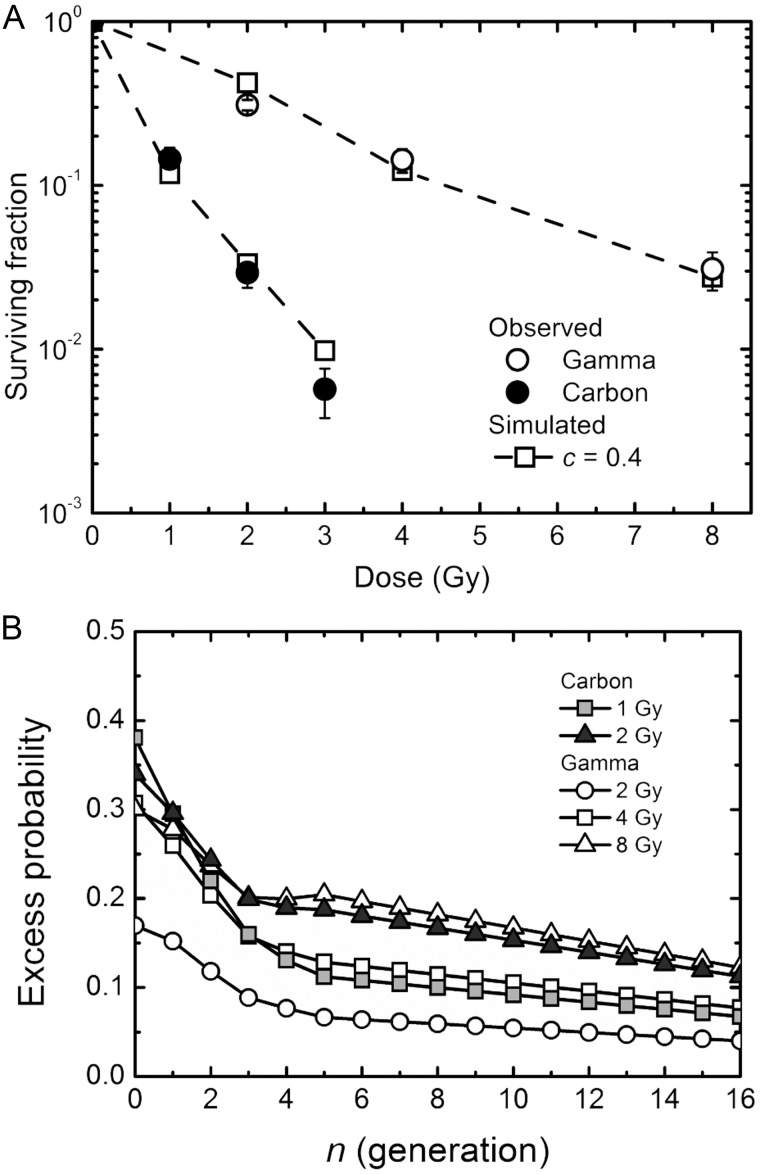
Comparison between low-LET γ-rays and high-LET carbon ions. (**A**) The observed surviving fraction (circles) and simulated one (squares). The data for γ-irradiation were taken from the previous report [7]. Circles indicate the experimentally determined surviving fraction, and lines with squares indicate the simulated surviving fraction with the specific slope *c* of 0.4 using the parameters obtained from the log–log fit. Error bars represent standard errors of the mean. (**B**) The excess reproductive cell death (RCD) probability. The data for 1 and 2 Gy of carbon ions and 2, 4 and 8 Gy of γ-rays were plotted.

RBE is an important index with to compare the effects of various types of radiation with different radiation quality. The RBE determined here by simulations was 3.5, which was very close to the experimentally determined RBE of 3.9 [3]. To understand the meaning of the RCD for the RBE, we investigated the temporal kinetics of the excess RCD probability. Intriguingly, cells exposed to 1 Gy of carbon ions and 4 Gy of γ-rays, and cells exposed to 2 Gy of carbon ions and 8 Gy of γ-rays showed the same trend in terms of the temporal kinetics of excess RCD probabilities (Fig. 7B). These results again suggest a strong relation between the long-lasting RCD and the surviving fraction.

### Secondary colonies

**Figure 8:**
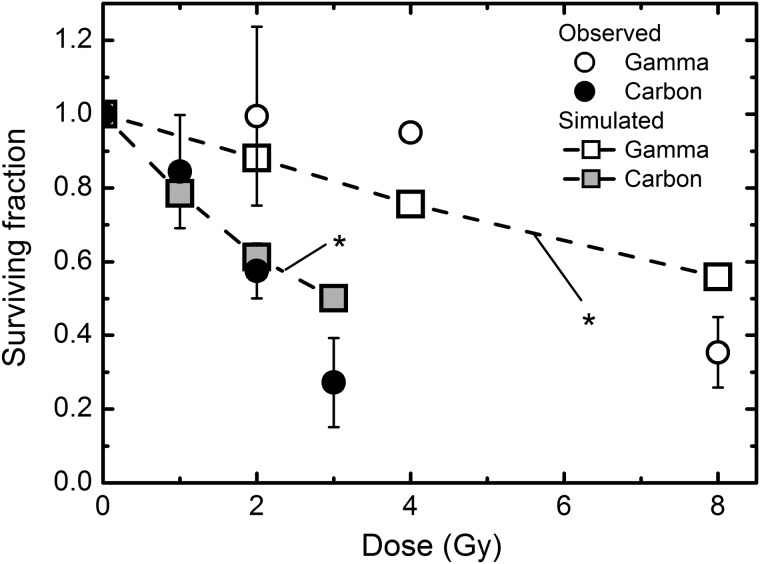
Surviving fraction of secondary colonies. The data for the experimentally determined surviving fraction were taken from the previous report [3]. The constant RCD probability for the secondary colony formation was assumed as the RCD probability for each dose at the 16th generation in primary colonies (Fig. 6B). Squares were estimated from the simulations of abortive colonies. Error bars represent standard errors of the mean. Asterisks indicate significant differences (*P* < 0.05) between observed and simulated dose–response curves tested by the chi-squared test with the Bonferroni correction.

We previously conducted formation of secondary colonies derived from cells in primary colonies, and found delayed RCD in such secondary colonies [3]. The secondary colony formation can be simulated *in silico* using the hypothetical RCD probability. For this, the constant RCD probability for the secondary colony formation was assumed to be the RCD probability for each dose at the 16th generation in primary colonies (Fig. 7B). Figure 8 shows the surviving fraction of the secondary colonies that were derived from primary colonies arising from fibroblasts exposed to γ-rays and carbon ions. Despite significant differences between the observed and simulated surviving fraction of the secondary colonies (Fig. 8), the simulated dose–response curves for γ-rays and carbon ions in the secondary colony formation well reflected the trend in the experimentally observed ones. These results demonstrate that the estimated RCD probability at the 16th generation was suitable for long-lasting RCD as an approximate range.

## DISCUSSION

To investigate the production kinetics of abortive colonies arising from normal human fibroblasts irradiated with high-LET carbon ions, we analyzed the colony size distribution and surviving fraction using a branching process model and a 2D colony expansion model. The present study provides insights into the colony production kinetics following exposure to low- and high-LET radiation.

The unique strength of our study was the ability to accurately simulate the RCD and the IR-induced excess RCD probabilities with our branching process model, although the experimental observation of RCD itself is experimentally well known. The present study showed the estimation of the persistent excess RCD probability up to 16 generations. It has also been reported that the RCD can persist for 12–23 generations after exposure [10], and that γ-H2AX foci remain 2 weeks following X-irradiation [11]. Though the mechanisms behind persistent RCD are not well understood, it is tempting to speculate on them. Genomic instability arises in the progeny of irradiated cells, whereas bystander effects occur in non-irradiated cells that have received signals from irradiated cells [12]. There is experimental evidence that the progeny of irradiated cells causes biological effects in bystander cells, and that the progeny of bystander cells undergo genomic instability [13]. Such interrelations between bystander effects and genomic instability indicate that continuous spatio-temporal propagation of signals initially transmitted from irradiated cells may perpetuate radiation effects in their neighborhood and progeny over time [13]. Thus, the persistent RCD may result from long-lasting non-targeted effects that activate signaling cascades (e.g. intra- or intercellular signaling leading to intra- or intercolonial signaling) [14]. The incorporation of the underlying mechanism into our model will be the next challenge.

Various events occur during the formation and analysis of colonies, such as a decrease in the number of cell divisions due to contact inhibition, delay of cell cycle progression, and loss of the inactivated/dead cells before the cell/colony count. In this regard, our 2D colony expansion model has taken account of contact inhibition, and was indeed capable of simulating the growth curve even in its plateau phase [7]. Events in which a cell cycle delay continues over 2 weeks during colony formation were judged as RCD in our present framework, and this was also the case for the colony formation assay such that cells undergoing such a long-term cell cycle delay should be regarded as non-survivors. Cell loss before the cell/colony count should partially be reflected in the plating efficiency, but such entire events were not evaluated in our analysis, necessitating further studies. Moreover, fate tracing of a parental cell directly exposed to IR and its daughter, granddaughter or further progeny cells would be critical for understanding the production kinetics of abortive colonies and persistent non-targeted effects. For example, time-lapse imaging is a powerful tool to trace the death of individual cells [15]; nevertheless, even with this modern technique, it would be difficult to trace the behavior of all cells during the whole colony formation process, because the traced cells are occasionally lost from the field of view [15]. Therefore, further development of time-lapse imaging is clearly necessary. On the other hand, a conventional colony formation assay is a feasible and easy technique. To evaluate the fates of parental cells and their progeny, the analysis of the primary and secondary colonies in combination with a branching process model analysis would be useful, as was done in this study.

**Figure 9:**
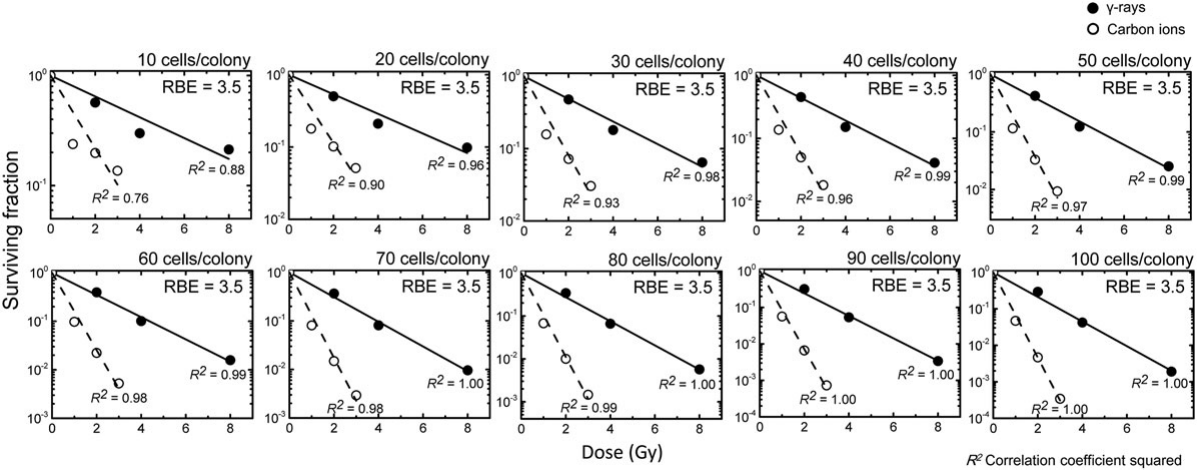
Dependence of the relative biological effectiveness (RBE) values on the threshold number of cells per colony as a criterion to distinguish between clonogenic and abortive colonies. Presented are the RBE values calculated using the criteria of 10, 20, 30, 40, 50, 60, 70, 80, 90 or 100 cells/colony (these numbers shown in the upper right in each panel). The surviving fraction calculated with the Monte Carlo method was fitted to the equation *y* = *exp* (*− ax*), where *a*, *x*, *y* are slope, dose and surviving fraction, respectively, and *R*^2^ denotes the correlation coefficient squared. Solid lines with solid circles indicate the surviving fraction for γ-rays, and dotted lines with open circles indicate the surviving fraction for carbon ions. The 10% survival dose was calculated as [*ln* (1/0.1)]/*a*, and the RBE was calculated based on the 10% survival dose.

The RBE such as that based on the iso-survival dose is a good indicator for understanding the dependence of biological effects on radiation quality. In this regard, our present finding that RCD in later generations influences the surviving fraction raises the question of how RBE changes when a colony size other than 50 is employed as the criterion to distinguish between clonogenic and abortive colonies. We could not test this in detail, because of the lack of datasets for the precise fraction of each colony containing 50 or more cells (e.g. the fraction of colonies each with 50 cells, 51 cells, 52 cells …). Interestingly, our rough estimate showed little if any difference in RBE values based on the 10% survival dose when the criterion of 10–100 (estimated at 10-cell intervals such as 10, 20, 30 …) cells/colony was used; however, it should be noted that the smaller the threshold number of cells per colony used as a criterion, the less linear the relationship between the dose and the surviving fraction on the semilog plot (Figure 9). Thus, neither the RBE nor the surviving fraction should be estimated based on a small threshold number (e.g. 10 cells/colony).

Secondary colonies derived from cells in primary colonies are reflective of the delayed RCD [3], and this was also supported by simulation of the secondary colony formation. Our findings suggest that the persistent RCD changes with generations, and that the RCD in later generations influences the surviving fraction. Although DNA damage and repair immediately following irradiation are important, we should keep in mind that events arising generations after irradiation can affect the surviving fraction. Further studies should elucidate the precise mechanisms underlying the persistent RCD. For example, there are two methods for characterizing the long-lasting RCD probability using a clonogenic assay. One is to measure the colony size distribution of colonies with 16 or more cells, but there are some difficulties (e.g. low frequency of large colonies, and an effort-consuming task for the enumeration of colonies). The other method is to perform secondary or tertiary colony formation. Combination of these two methods can provide important information on the delayed RCD probability. In addition, an application of molecular analysis to a subsequent colony formation assay may play an important role in understanding the late occurring effects in the descendants of irradiated cells. In fact, as shown in Fig. 8, simulations underestimated the surviving fraction for carbon ions and overestimated it for γ-rays due to the shoulder in the survival curve, implying that the RCD probability in the secondary colony is partly controlled by the repair system of a cell. However, the trend in Fig. 8 is not simple: the relationship between the experiment and the simulation differs at low dose range compared with the high dose range, encouraging further analysis (e.g. of abortive colonies in secondary colonies).

## CONCLUSION

In conclusion, our present framework for a colony formation assay is applicable to low- and high-LET irradiated cells, and provides a persistent RCD probability. Our findings suggest that the long-lasting RCD is an important determinant of the surviving fraction and its RBE.
